# Landmark-Based Drift Compensation Algorithm for Inertial Pedestrian Navigation

**DOI:** 10.3390/s17071555

**Published:** 2017-07-03

**Authors:** Estefania Munoz Diaz, Maria Caamano, Francisco Javier Fuentes Sánchez

**Affiliations:** German Aerospace Center (DLR), Institute of Communications and Navigation, Oberpfaffenhofen, 82234 Wessling, Germany; Maria.CaamanoAlbuerne@dlr.de (M.C.); franciscojavier.fuentessanchez@gmail.com (F.J.F.S.)

**Keywords:** Landmark, inertial, pedestrian, navigation, pocket, drift, yaw, corners, stairs

## Abstract

The navigation of pedestrians based on inertial sensors, i.e., accelerometers and gyroscopes, has experienced a great growth over the last years. However, the noise of medium- and low-cost sensors causes a high error in the orientation estimation, particularly in the yaw angle. This error, called drift, is due to the bias of the z-axis gyroscope and other slow changing errors, such as temperature variations. We propose a seamless landmark-based drift compensation algorithm that only uses inertial measurements. The proposed algorithm adds a great value to the state of the art, because the vast majority of the drift elimination algorithms apply corrections to the estimated position, but not to the yaw angle estimation. Instead, the presented algorithm computes the drift value and uses it to prevent yaw errors and therefore position errors. In order to achieve this goal, a detector of landmarks, i.e., corners and stairs, and an association algorithm have been developed. The results of the experiments show that it is possible to reliably detect corners and stairs using only inertial measurements eliminating the need that the user takes any action, e.g., pressing a button. Associations between re-visited landmarks are successfully made taking into account the uncertainty of the position. After that, the drift is computed out of all associations and used during a post-processing stage to obtain a low-drifted yaw angle estimation, that leads to successfully drift compensated trajectories. The proposed algorithm has been tested with quasi-error-free turn rate measurements introducing known biases and with medium-cost gyroscopes in 3D indoor and outdoor scenarios.

## 1. Introduction

There is nowadays a high demand for pedestrian navigation systems. Many of them are integrated in safety-of-life services such as disaster management for rescue personnel. Pedestrian navigation systems, however, are not only restricted to the professional market. Their demand is widespread for all kind of location-based services such as guidance in airports, hospitals or shopping malls. A classic solution is to integrate the pedestrian navigation system in the smartphone that, among others, has inertial sensors embedded. The so-called inertial sensors, i.e., accelerometers and gyroscopes, are usually based on micro electromechanical (MEMS) technology. MEMS are widely used due to their miniaturization and prize reduction.

The navigation based on inertial sensors can be split into two steps: displacement and orientation estimation. The displacement estimation requires for pedestrian navigation first detect the steps and then estimate the travelled distance between steps, horizontally and vertically. The orientation estimation, which is briefly explained in the following, can be expressed as Euler angles, i.e., roll, pitch and yaw. The orientation estimation represents the major contribution to the error in position. Although many publications have been written about pedestrian inertial navigation systems using medium- and low-cost MEMS sensors, some issues are not yet completely solved, such as the accumulated error in the yaw angle estimation. This error, commonly called drift, should be computed and used to prevent positioning errors. In order to obtain the orientation of the sensor, the turn rate measurements provided by the three mutually orthogonal gyroscopes have to be integrated once with respect to time. However, the above mentioned turn rate measurements contain also biases. The bias of a gyroscope is the averaged value of the turn rate measurements during a static phase. The integration of a constant bias causes an error growing linearly with time. The bias stability describes how the gyroscope’s bias may change over a specific period of time under stable conditions, usually at a constant temperature. Temperature fluctuations due to changes in the environment and sensor self-heating modify the bias value. Therefore, the Euler angles computation consisting solely on the integration of turn rate measurements is erroneous due to biases and other slow changing errors [1]. The biases of the x- and y-axes gyroscopes can be estimated through the gravitational field [2]. Therefore, the error in roll and pitch angles can be corrected with a successfully estimation of the x- and y-axes biases. On the contrary, the yaw angle suffers from ever growing errors that mainly arise from a poor estimation of the bias of the z-axis gyroscope [2]. Indeed, the yaw angle estimation is of key importance to the estimation of the trajectory, because the yaw angle represents the heading of the user. The main consequence of the presence of drift is a gradually twisted trajectory. In fact, this is our main motivation to research a drift compensation algorithm that is able to correct the yaw angle error.

There are many methods in the literature to correct the drift error, mainly based on the fusion of inertial measurements and other sources of information like WLAN/UWB ranges or satellite pseudoranges, among others. However, the fusion of the positioning information stemming from different sensors, like previously mentioned, is out of the scope of this article. In the following, the literature regarding alternative drift correction algorithms is exposed:

### 1.1. Heuristic Drift Elimination Algorithms

Many heuristic drift elimination algorithms have been suggested in the literature for pedestrian indoor positioning. These algorithms assume that pedestrians walk on a straight line within the building in directions which are parallel to the outer walls of the building. If the navigation system detects that the pedestrian does not move on a straight line, these corrections are suspend [3]. After the heuristic drift elimination algorithm was first published, many authors in the literature have proposed similar ideas or improvements, such as coping with complex buildings including curved corridors or wide areas not restricted by corridors [4,5,6].

Additionally, the heuristic drift elimination has been suggested in combination with other heading corrections such as zero angular rate updates and magnetic measurements [7,8]. The combination with available maps has also been proposed to restrict the possible heading angles by taking into account the walls of the buildings [9,10]. The high non-linearities of the maps force the use of particle filters that weight the particles according to the similarity of their heading with the direction of the walls. The main drawback of these approaches is that previous knowledge is necessary, e.g., the map or the shape of the corridors.

### 1.2. Landmark-Based Algorithms

In [11], a study has been carried out concluding that landmarks play an important role for pedestrian navigation, therefore, it is recommendable to develop methods to include landmarks information in pedestrian navigation systems.

One of the most intuitive ways of detecting landmarks within the trajectory described by the pedestrian is using visual information. This information derived from the chosen landmarks is tracked over time in order to use this motion to constrain the drift. In [12], a stereo vision camera is used to extract the optical information of the landmarks. The heuristic drift elimination algorithms can also be seen as landmark-based algorithms, since the manmade straight corridors can be interpreted as landmarks. The main difference is that the landmarks of the heuristic drift elimination algorithms do not need to be tracked over time.

In [13], an algorithm that makes use of detected ramps in buildings for correcting the drift is presented. In the article, foot-mounted inertial sensors are used and the position of the ramps of the target building is previously known. Ramps are detected through the slope of the terrain and corrections of the position of the pedestrian are applied. However, this approach does not compute the drift value, therefore, although the position is corrected, the proposed approach does not bound the error of the yaw angle.

### 1.3. SLAM-Based Algorithms

A suitable solution to drift reduction is the use of the simultaneous localization and mapping (SLAM) algorithm, which has been used for decades in robotics. The SLAM algorithm simultaneously generates a map of the desired landmarks and locates the user/robot within this map. These landmarks can be detected with any sensor, such as a laser scanner or a camera. The automatic vacuum cleaner, for example, generates a map of the room where the interesting landmarks, i.e., sofa, table, doors, are included and it locates itself within this map.

The SLAM algorithm has also be adapted to pedestrian navigation aiming at reducing the drift error. In order for the SLAM algorithm to successfully reduce the drift, a “loop closure” is necessary. That means, the pedestrian detects landmarks during the trajectory and, when part of the trajectory is re-visited, the landmark is again detected. The same landmark detected twice is an indicative of being again at the same position, therefore, corrections can be applied.

Commonly a particle filter is used that generates particles that move with different errors. When landmarks are re-visited, all particles are weighted depending on the landmarks position. Thus, particles that followed a trajectory with the current drift are high weighted, because they most likely correspond to the detected position. In [14], the 2D space is divided into a grid of uniform and adjacent hexagons, which can be considered as landmarks. The pedestrian visits these hexagons while walking and, when the loop is closed, the same hexagons are re-visited leading to the aforementioned corrections. The same procedure is applied for 3D trajectories but dividing the volume into hexagonal prisms with eight faces [15]. This procedure can also be applied if the chosen landmarks are adjacent hexagons identified by the magnetic field intensity measured with a magnetometer while the pedestrian is walking [16]. The main drawback of these algorithms is the complexity and processing time to manage the numerous hexagons or hexagonal prisms.

In [17], the proposed landmarks are some location-related activities carried out by the pedestrian, such as sitting, lying or opening doors, among others. Based on the assumption that these activities are always performed at the same place, their repeated detection leads to the aforementioned corrections. The main drawback of the particle filter approach is that heading corrections are usually not fed back to the orientation estimation filter, thus further drift and positioning errors are not prevented.

The objective of this paper is to present a seamless drift compensation algorithm based on landmarks that only uses inertial measurements. This algorithm is able to compute the accumulated drift error and use it to successfully generate a low-drifted yaw angle estimation and drift compensated trajectories in a post-processing stage. To that end, a landmark detection algorithm based on inertial measurements is presented. The detection is seamlessly done, since the user does not have to take any action when reaching a new landmark, i.e., pressing a button. The selected landmarks are corners and stairs, which are stored in a data base and used when the pedestrian re-visits them. After that, the landmark associator, which takes into account the uncertainty of the position estimation, is detailed. Then the computation of the accumulated drift error and the post-processing drift compensation are presented. The proposed algorithm, presented in Section 2, is tested in real 3D scenarios and the results are summarized in Section 3.

## 2. Proposed Landmark-Based Drift Compensation Algorithm

The proposed landmark-based drift compensation algorithm enhances the inertial pocket navigation system presented in [18]. First, the orientation is computed using an unscented Kalman filter whose states are the Euler angles roll ϕ, pitch θ, yaw ψ and the biases of the gyroscopes $b_{x}$, $b_{y}$ and $b_{z}$, as explained in [2]. The detection of the successive maximums of the pitch angle estimation is used for the displacement estimator in order to estimate the step length *s* and the vertical displacement *v* stepwise [18]. Finally, the 3D position (x,y,z) is calculated with the step length, the vertical displacement and the yaw angle estimation as follows:

(1) $$\begin{array}{cc} x_{k} & =x_{k-1}+s_{k}\;\cdot \;\cos\left(\psi_{k}\right),\\ y_{k} & =y_{k-1}+s_{k}\;\cdot \;\sin\left(\psi_{k}\right),\\ z_{k} & =z_{k-1}+v_{k}. \end{array}$$

**Figure 1:**
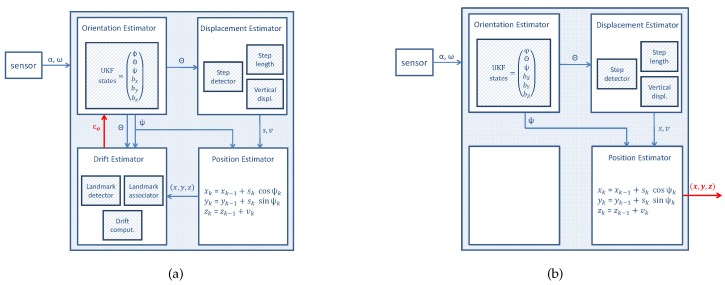
Proposed enhanced inertial pocket navigation system by including the landmark-based drift compensation algorithms in two steps: (**a**) first the drift value $\epsilon_{0}$ is computed and (**b**) second this value is used by the orientation estimator to generate a drift compensated trajectory.

In order to estimate the drift, we propose to use the pitch and the yaw angles to detect landmarks during the walk. These landmarks are associated, if repeated, and the drift error accumulated between them is computed. The resulting drift $\epsilon_{o}$ is the output of the first stage as shown in Figure 1a. In the post-processing stage, $\epsilon_{o}$ is used by the orientation estimation filter to generate a drift-reduced yaw angle that yields a drift compensated trajectory (Figure 1b).

Indeed, we assume that the drift error is constant and this assumption does not hold for long periods. However, with this work we want to provide the proof of concept that it is possible to compute the drift error and use it in the orientation estimator filter to obtain a drift-reduced yaw angle. Thus, the proposed method could be used to apply drift corrections online, being so adapted to drift changes over time.

Landmarks should be easily observable and re-observable, distinguishable from each other, stationary and plentiful in the environment. Taking into account this, we have selected our landmarks based on the fact that, although the movement of the pedestrian is to some extent unconstrained, typical manmade scenarios introduce some restrictions. Due to the layout of buildings and cities, the most common corner in rooms and between building blocks describes a 90${}^{\circ }$ turn. Likewise, the stairs are always present as elements for connecting different floors. Therefore, we will use corners and stairs as landmarks. In order to properly describe the landmark detection and association algorithms, the position of the landmarks is necessary. The position of the landmark is coincident with the position of the pedestrian at the moment of the detection.

### 2.1. Landmark Detection

The landmark detection algorithm identifies the aforementioned landmarks, i.e., corners and stairs, automatically while the pedestrian walks. The drift compensation algorithm we propose makes only use of inertial sensors, thus the barometer will not be used to detect stairs. We solve the detection of corners and stairs using the yaw and pitch angles, respectively.

#### 2.1.1. Corners

**Figure 2:**
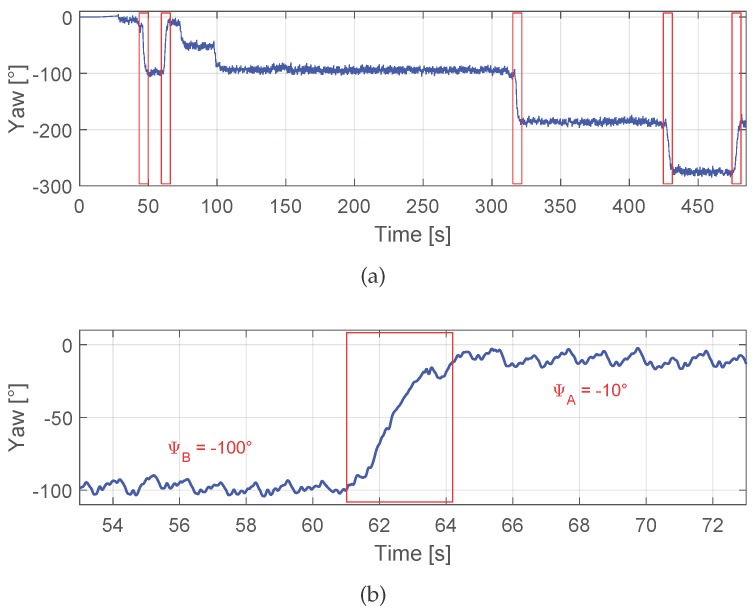
The blue curve represents the yaw angle estimation during a walk where the inertial sensors are placed in the front pocket of the trousers. The detected corners are highlighted in red. (**b**) depicts a zoom of the second corner of (**a**).

The proposed algorithm identifies the corners, defined in this work as 90${}^{\circ }$ turns, using the yaw angle estimation. Figure 2a shows in blue the yaw angle estimation derived when the inertial sensors are placed in the front pocket of the trousers. Initially, the pedestrian was standing for almost 25 s. If the pedestrian walks straight, the yaw angle estimation is stable. The oscillation zoomed in Figure 2b corresponds to the movement of the hip of the pedestrian while walking. When the pedestrian turns around a corner, the yaw angle estimation changes suddenly from the previous heading $\psi_{B}$ to the new one $\psi_{A}$. The corner detector algorithm identifies these periods when the stability changes. The red rectangles highlight the yaw angle estimation during the detected corners corresponding to 90${}^{\circ }$ turns, which are identified by the corner detector. Between second 75 and 100, two consecutive 45${}^{\circ }$ turns are made, but these are not considered by the corner detector algorithm.

Two buffers whose window length is *q* are used to detect corners using the yaw angle estimation:

(2) $$\begin{array}{cc} \psi_{B} & =\frac{1}{q}\;\cdot \;\sum_{k=1}^{q}\psi_{k},\\ \psi_{A} & =\frac{1}{q}\;\cdot \;\sum_{k=q+w}^{2q+w}\psi_{k}, \end{array}$$

where $\psi_{k}$ is the yaw angle estimation at the time stamp *k*, $\psi_{B}$ and $\psi_{A}$ represent the averaged yaw angle before and after the corner, respectively, and *w* stands for the separation between buffers.

The movement of the hip makes the use of a buffer necessary to compute the averaged yaw angle value over a significant period, instead of taking a single value before and after the corner. Given that a typical walking speed is one step per second, the window length is chosen to be 1 s. Therefore, $q=1\;\cdot \;f_{s}$, being $f_{s}$ the sampling frequency of the sensor. The separation between the averaged yaw angle before and after the corner, $\psi_{B}$ and $\psi_{A}$ respectively, is set to 5 s. Therefore, $w=5\;\cdot \;f_{s}$. The values of *q* and *w* will be initially set as indicated, however, there is a strong dependency on the walking speed. Thus, the values of *q* and *w* will be adapted taking it into account. Therefore if the speed decreases, the values of *q* and *w* are correspondingly enlarged. The walking speed of the pedestrian is computed based on the step length and the time between detected steps, algorithms presented in [19].

**Figure 3:**
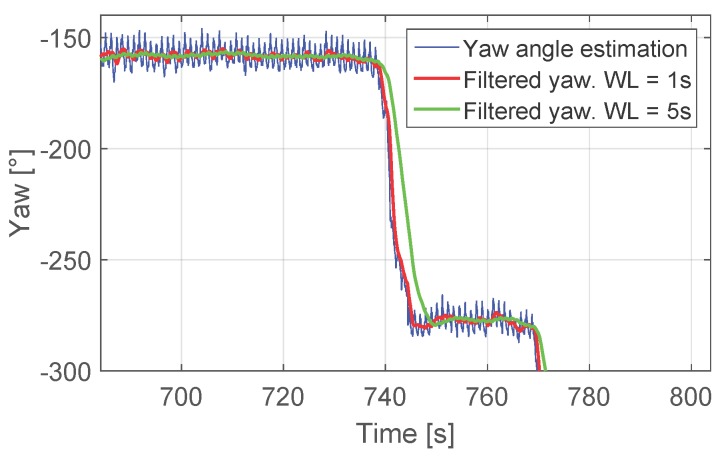
The blue curve represents the yaw angle estimation, the red curve is the filtered yaw angle estimation with a window length of 1 s and the green curve is the result of filtering the yaw angle with a window length of 5 s.

As Figure 3 shows, the yaw angle estimation contains peaks of approximately 20${}^{\circ }$ amplitude for a comfortable walking speed. These peaks do not affect the corner detection, but the values $\psi_{A}$ and $\psi_{B}$. The window length of the buffer *q* has been set to 1 s in order not to delay the corner detection. However, as seen in the figure, a window length of 5 s offers a more stable filtered yaw angle.

Medium-cost MEMS gyroscopes have a short-term stability and turns around corners are usually made within a very short period of time. Thus, the estimated jump in the yaw angle during corresponding to the corner is considered error-free. However, the natural walking does not always describe exact 90${}^{\circ }$ turns. Thus, a corner is detected when the absolute value of the difference of the averaged yaw angles $\psi_{B}$ and $\psi_{A}$ lies between $r_{1}$ and $r_{2}$ according to:

(3) $$r_{1}<\left|\psi_{B}-\psi_{A}\right|<r_{2},$$

where $r_{1}=$ 80${}^{\circ }$ and $r_{2}=$ 100${}^{\circ }$. The difference between the averaged yaw angle before and after the corner is evaluated constantly and regardless its sign. The sign of the yaw angle estimation responds to the direction of the turn, thus a turn to the right will increase the yaw angle value and a turn to the left will decrease it. The corner detector identifies corners described in both directions.

#### 2.1.2. Stairs

Stairs represent an unambiguous landmark that connects different floors. The proposed detector algorithm identifies the stairs using the pitch angle estimation based on the fact that, from the biomechanical point of view, the aperture angle of the leg changes between walking horizontally and walking up- and downstairs. The algorithm of the stairs detector has been presented in [18]. The detection of the physical activities walking up- and downstairs corresponds to the detection of the stairs.

### 2.2. Landmark Association

In order to associate re-visited landmarks, first we store them in a data base at the moment of the detection. The landmark data base, whose structure is shown in Table 1, comprises the parameters of the landmarks. The table of the example has two landmarks stored, L1 and L2. The *Type* flag is 0 for corners and 1 for stairs. This flag is designed to reduce the searching time, since it allows comparing only landmarks of the same type. The *Time* is the time stamp where the landmark has been detected, counting from the beginning of the walk. The *Position* (x,y,z) is the 3D position of the landmark in the navigation frame, which is stored in the data base together with its *Uncertainty*, which is the variance-covariance matrix representing the position uncertainty. Last, the *Yaw* angle value is stored and has two formats: for corners it defines the yaw angle before $\psi_{B}$ and after turning the corner $\psi_{A}$ (see Figure 2b), and for stairs it defines the direction in which the stairs are gone through.

The decision whether two landmarks can be associated is taken based on the position of the current landmark and the position of all stored landmarks of the same *Type* detected on the same floor. The position, however, will not be coincident even for the same landmarks due to the accumulated drift over time and also due to other possible errors in the step length computation or false step detections, among others.

**Figure 4:**
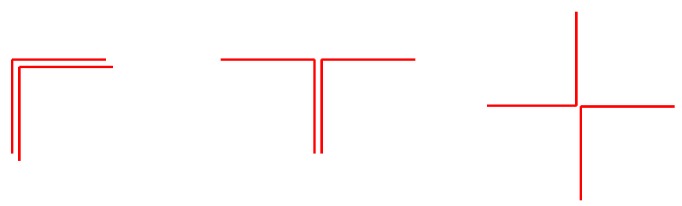
Different corner combinations that are associated by the corner associator regardless of the direction of the trajectory.

The associator does not differentiate the direction in which the stairs are gone through. Therefore, a staircase will be associated if the first time the stairs were climbed up and the second time were climbed down, as well as if it is both times climbed up or climbed down. The same applies to corners, that are associated regardless of the direction of the trajectory, which causes different *Yaw* combinations. Figure 4 shows the different corner combinations that the associator matches: trajectory completely overlapped, trajectory partially overlapped and no overlap.

There are different metrics to evaluate the distance between landmarks. The Euclidean distance has been discarded because it does not account for the uncertainty, which is an ellipsoid shaped volume around the mean value of the position. The Mahalanobis distance [20] is more adequate to evaluate the distance between landmarks:

(4) $$\Delta_{1,2}^{2}=\Delta_{2,1}^{2}=\left[\left(x_{1},y_{1},z_{1}\right)-\left(x_{2},y_{2},z_{2}\right)\right]\;\cdot \;{\left[P_{1}+P_{2}\right]}^{-1}\;\cdot \;{\left[\left(x_{1},y_{1},z_{1}\right)-\left(x_{2},y_{2},z_{2}\right)\right]}^{T},$$

where $\Delta_{1,2}$ accounts for the distance between the stored and the current landmark, $\left(x_{i},y_{i},z_{i}\right)$ for i={1,2} represents the position of each landmark and $P_{i}$ for i={1,2} represents the variance-covariance matrix of each position.

For multivariate normally distributed data, the squared Mahalanobis distances $\Delta^{2}$ are approximately χ-square distributed with *n* degrees of freedom. For the algorithm proposed in this article, n=6, being the 6 degrees of freedom of the 3D position of the pedestrian: (x,y,z), the yaw angle ψ, the step length *s* and the vertical displacement *v* (see Figure 1). The threshold κ to decide if the current landmark is possibly the same as one of the stored ones is fixed and also depends on the confidence level. For a confidence level of 99.7 % and n=6 the threshold κ=4.48 [21].

Associations are therefore decided taking into account the uncertainty of the position, which includes the uncertainty of both, heading and displacement. Thus, if the uncertainty is large due to the accumulated drift error, more than one hypothesis might be possible. The maximum elapsed time to associate landmarks strongly depends on the heading uncertainty and on the distribution of the detected landmarks. A bias value of 0.1${}^{\circ }$·s${}^{-1}$ causes 360${}^{\circ }$ error in the heading estimation after 1 h. This rapid growth of the heading error due to the bias, makes it important to have drift observations in early phases and ideally apply these corrections online.

### 2.3. Drift Computation

**Figure 5:**
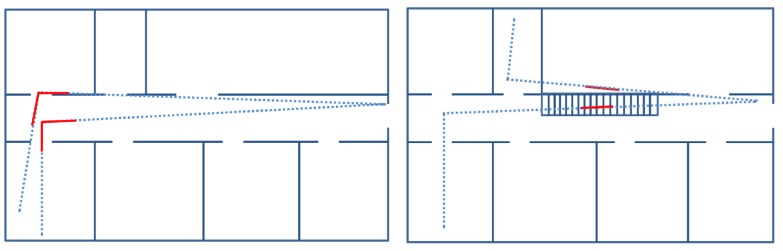
Schematic drifted trajectory including corners (**left**) and stairs (**right**).

The drift computation algorithm for corners and stairs is based on the principle that having zero drift, the yaw angle values of associated landmarks are equal. Figure 5 (left) shows a schematic drifted trajectory represented with the dashed blue line. The pedestrian left the room, walked to the main entrance and went back to the same room. A corner, highlighted in red, is detected when leaving and entering the room. These corners are identified as the same landmark. Figure 5 (right) shows a schematic 3D drifted trajectory represented with the dashed blue line. In the represented walk, the pedestrian left the first room, climbed up the stairs to the second floor and then climbed down the stairs and entered the small room on the other side of the building. The stairs, highlighted in red, are detected when going up and down. Both parts of the trajectory described on the stairs will be associated as the same landmark.

**Figure 6:**
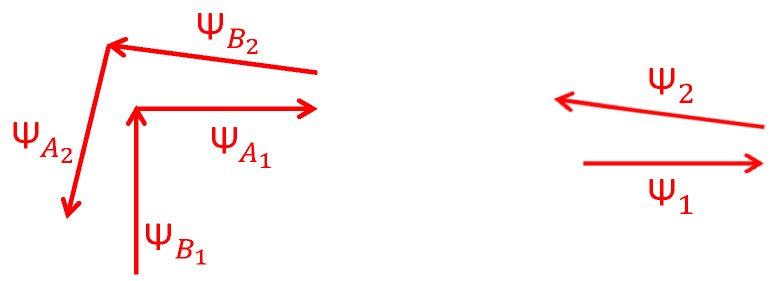
Yaw angle labels for the landmarks corner and stairs represented respectively in Figure 5.

The red arrows in Figure 6 represent the landmarks identified in Figure 5. For the case of the corner represented in Figure 6 (left), the first time the landmark is detected is labeled with the sub-index 1 and when it is re-visited the sub-index is 2. As already explained, the corners are composed of two yaw angles: before and after turning the corner. For the case of the stairs represented in Figure 6 (right), the yaw angle measured the first time the landmark is detected is labeled with the sub-index 1 and when the landmark is re-visited the sub-index used is 2.

The deviation angle δ represents the mismatch of trajectories, measured in degrees, due to the drift error. The angle δ is computed by subtracting the yaw angle stored in the data base when the landmark was for the first time visited and the currently detected yaw angle. For Figure 6 (left) the deviation angle is computed as follows:

(5) $$\begin{array}{cc} \delta_{1} & =\psi_{B_{1}}-\psi_{A_{2}},\\ \delta_{2} & =\psi_{A_{1}}-\psi_{B_{2}},\\ \delta & =\frac{\delta_{1}+\delta_{2}}{2}. \end{array}$$

In the case of the stairs, as represented in Figure 6 (right), the deviation angle is computed as follows:

(6) $$\delta =\psi_{1}-\psi_{2}.$$

The deviation angle should be, in the absence of drift, 0${}^{\circ }$ or 180${}^{\circ }$ if the landmark has been visited in the same or in opposite direction, respectively.

In a general case valid for corners and stairs, the observations, which are the yaw angles detected the first visit and the re-visit, $\psi_{1}$ and $\psi_{2}$, are related to the drift value ϵ as indicated:

(7) $$\psi_{2}=\psi_{1}+\int_{t_{1}}^{t_{2}}\;\epsilon \;dt.$$

In the case of constant drift:

(8) $$\epsilon =\frac{\delta }{\Delta t}$$

being ϵ the drift, δ the deviation of the trajectory and $\Delta t=t_{2}-t_{1}$ the elapsed time between the first visit and the re-visit. Landmarks are not re-visited with exactly the same yaw angle, therefore we account for an error of 4${}^{\circ }$ standard deviation in $\psi_{1}$ and $\psi_{2}$, respectively. This error implies that the elapsed time between landmarks Δt should be larger than 2 min in order for the standard deviation of the resulting drift ϵ to be smaller than 0.05${}^{\circ }$·s${}^{-1}$. Associations whose elapsed time is shorter than 2 min are discarded.

### 2.4. Post-Processing Drift Compensation

The drift compensation occurs in two steps: First, the inertial measurements are analyzed in order to find and associate landmarks. With the associations made, the drift value is computed (Figure 1a). Second, the recorded inertial measurements are processed and the trajectory is computed by using the drift value $\epsilon_{o}$ in the initial configuration parameters of the orientation estimation filter. The readers are referred to [2] to know more details about the orientation estimation filter. Initially the biases of the gyroscopes are set to zero. Given that the drift is mainly caused by the bias in the z-axis, for the post-processing stage (Figure 1b), when the drift value is computed, the initial gyroscopes’ biases will be set as:

(9) $$(0,0,\epsilon_{o}).$$

The drift value ϵ is computed taken into account the drift values resulting from all associations made during the walk, but rejecting possible outliers. The decision whether the drift value is an outlier or not is based on the expected drift values given the sensor characteristics and the cohesion of all values obtained. The drift value is computed weighting the total number of associations *m* with the elapsed time between landmarks Δt.

(10) $$\epsilon_{o}=\frac{1}{\sum_{l=1}^{m}\Delta t_{l}}\;\cdot \;\sum_{k=1}^{m}\Delta t_{k}\;\cdot \;\epsilon_{k}.$$

The drift computation algorithm is more robust to errors if the elapsed time between associated landmarks is larger, as previously mentioned. Therefore associations with larger elapsed time between landmarks are more reliable.

## 3. Experimental Results

The algorithms proposed in this article that constitute the drift compensation algorithm, i.e., the landmark detector, the landmark associator and the drift computation algorithm, will be evaluated in this section. First, we will introduce known gyroscopes’ biases in order to have a controlled drift error. To do that, we will use quasi-error-free turn rate measurements recorded with the IMU DSP-1750 from KVH [22], which embeds fiber optic gyroscopes (FOG) and MEMS accelerometers. Second, to test the proposed drift compensation algorithm with MEMS gyroscopes with unknown biases and drift error, we will use the measurements recorded with the medium-cost MEMS IMU MTw from Xsens [23].

A 2D walk, that has been recorded by a 1.52 m height woman at the Theresienhöhe in Munich, will be used to experimentally study the relationship between drift and bias of the z-axis gyroscope. This is of high importance because the proposed algorithm is strongly based on the fact that the drift is mainly composed of the z-axis gyroscope’s bias (see Equation (9)). A 3D walk recorded by a 1.82 m height man at the Earth Observation Center building of the German Aerospace Center (DLR) will be used to test the stair detection, the corner detection for large curvature radius and the association of landmarks at different heights, i.e., associations between landmarks situated on different floors are discarded. In order to test the proposed drift compensation algorithm with measurements recorded by MEMS inertial sensors, we will use a 3D walk recorded by a 1.70 m height woman in the Deutsches Museum of the city of Munich. This walk includes stairs and corner of different angles of aperture.

### 3.1. Relationship Between Drift and z-Axis Gyroscope’s Bias

**Figure 7:**
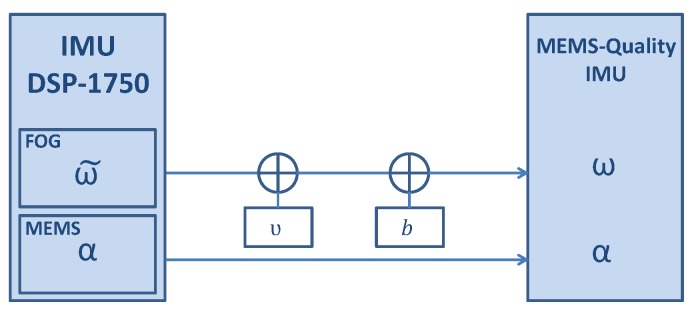
Diagram of the IMU DSP-1750 with the post-processing to generate MEMS-quality turn rate measurements.

This analysis has been conducted in order to experimentally evaluate how the z-axis gyroscope’s bias influences the drift error. In order to accurately know the z-axis gyroscope’s bias value, we have used the IMU DSP-1750, which contains FOGs as aforementioned. The turn rate measurements of the FOGs will be considered as quasi-error-free in this work. Readers are referred to [2] for the complete justification of treating the FOG turn rate measurements as quasi-error-free. The IMU DSP-1750 has been used to record the measurements and these have been post-processed in order to obtain MEMS-quality turn rate measurements with known bias values as indicated in Figure 7 and Equation (11):

(11) $$\omega =\tilde{\omega }+\nu +b,$$

being $\tilde{\omega }$ the quasi-error-free turn rate, ν white Gaussian noise, b the biases and ω the resulting MEMS-quality turn rate. The noise values are based on the MEMS MTw gyroscopes and are 0.1${}^{\circ }$·s${}^{-1}$ for the noise and (0,0,0.05)${}^{\circ }$·s${}^{-1}$ for the biases of the x-, y- and z-axes gyroscopes, respectively.

**Figure 8:**
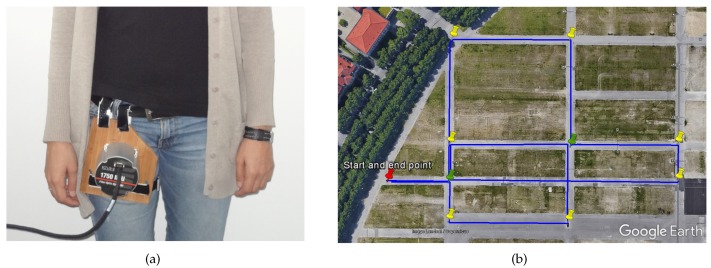
(**a**) IMU DSP-1750 test set up. (**b**) trajectory of the experiment represented with a blue line. The pins highlight the corners described during the walk.

The IMU DSP-1750 has been mounted externally on the upper part of the leg of the pedestrian with the help of a solid wood base, as indicated in Figure 8a. The experiment, whose trajectory is represented in Figure 8b with a blue line, has a duration of 22 min, consists of a path of approximately 1.1 km long and has been performed covering an area of approximately 46,000 m${}^{2}$ at the Theresienhöhe in Munich.

**Figure 9:**
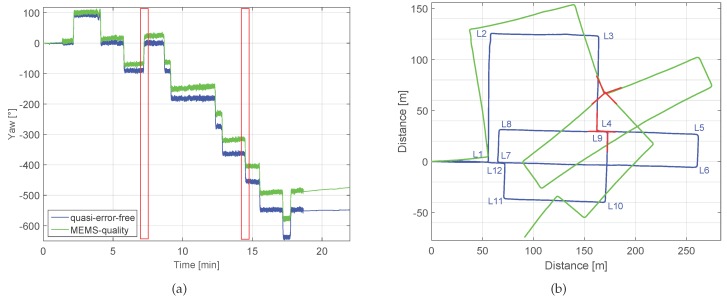
(**a**) represents the yaw angle estimation and (**b**) the trajectory of the walk corresponding to this experiment. The blue curves have been computed using the quasi-error-free turn rate measurements and the green curves have been computed with the MEMS-quality turn rate measurements. The corners are labeled.

Once the experiment has been recorded, the post-processing stage indicated in Figure 7 leads to the results shown in Figure 9. The yaw angles shown in Figure 9a have been computed integrating the turn rate measurements without subtracting the biases estimation or applying corrections. The blue curve shows the yaw angle using the quasi-error-free turn rate measurements and the green line has been computed using the MEMS-quality turn rate measurements. To obtain the trajectories of Figure 9b, the displacement estimation algorithms for the step detection and step length estimation presented in [18,19] have been used. The blue curve shows the yaw angle using the quasi-error-free turn rate measurements and the green line has been computed using the MEMS-quality turn rate measurements.

In order to find the drift value, we use the method proposed in this work. The walk contains 12 corners that have been labeled in Figure 9b. We choose for computing the drift the corners L4 and L9, highlighted in red, which are the case of no overlap shown in Figure 4 (right). First, we take from Figure 9a the *Yaw* and *Time* values of corners L4 and L9, highlighted with red rectangles. Table 2 summarizes this information:

The deviation angles, $\delta_{1}$ and $\delta_{2}$ are 22.7${}^{\circ }$ and 22.1${}^{\circ }$, respectively (see Equation (5)). Thus, δ is equal to 22.4${}^{\circ }$. This value represents the error that has been accumulated over $\left(t_{2}-t_{1}\right)$, thus Δt is equal to 437.8 s. Therefore the drift value ϵ extracted from the method proposed in this work is equal to 0.051${}^{\circ }$·s${}^{-1}$. Taking into account that the artificially introduced z-axis gyroscope’s bias $b_{z}$ is equal to 0.05${}^{\circ }$·s${}^{-1}$, it can be concluded that the drift is mainly composed of z-axis gyroscope’s bias under constant conditions, e.g., temperature.

In order to discard the possibility that biases of the x- and y-axes gyroscopes also affect the yaw angle estimation, and as a consequence, also the computed drift value after an association, we have computed the error of the yaw angle estimation caused by a x- and y-axes gyroscopes’ biases of 0.05${}^{\circ }$·s${}^{-1}$. The error has been computed by subtracting the resulting yaw angle estimation to the quasi-error-free yaw angle shown in blue in Figure 9a. In the case of a biases set of (0.05,0,0)${}^{\circ }$·s${}^{-1}$ the error caused on the yaw angle estimation is growing with time, reaching 5.5${}^{\circ }$ after 22 min. In the case of a biases set of (0,0.05,0)${}^{\circ }$·s${}^{-1}$ the error caused on the yaw angle estimation grows until 1${}^{\circ }$ after 22 min. The two aforementioned experiments yield however to severe errors in the roll and pitch angle estimations, respectively. Nevertheless, the x- and y-axes gyroscopes’ biases can be correctly estimated trough the gravity field (see [2]) and this estimation is used to correct the errors in roll and pitch angles. In this case, the remaining error transferred to the yaw angle is negligible. Therefore, we conclude that only the z-axis gyroscope’s bias affect substantially the underlying drift error.

### 3.2. Landmark Detector

The landmark detector will be tested for the corners using the drifted green trajectory depicted in Figure 9b. The 12 corners should be detected even in the presence of drift. Table 3 summarizes the automatically generated data base, where the uncertainty values have been omitted for conciseness.

For this experiment the walking speed was on average 3 km·h${}^{-1}$ and all corners are successfully detected. For higher walking speeds, the initially set values for the parameters *w* and *q* are also appropriate, because the yaw angle transition before and after the corner is steeper. Nevertheless, both parameters can be adapted based on the known walking speed. Additionally, the curvature radius of the corner plays an important role in the detection. For a large curvature radius, the detection might not be successful due to the smooth yaw angle transition before and after the corner. For these cases, the parameter *w* should be larger. However, there is no information available to adapt this parameter in the case of large curvature radii, since the trajectory is not previously known. Therefore, these corners are not detected and, consequently, not used for the drift computation.

**Figure 10:**
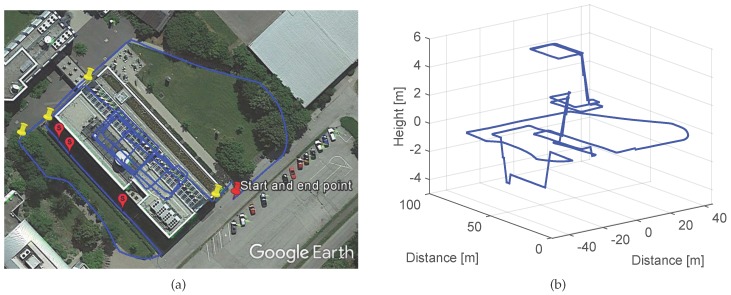
(**a**) shows the trajectory computed with the quasi-error-free measurements. The red pin highlights the start and end point, the yellow pins the detected outdoor corners and the pink placemarks the detected outdoor stairs. (**b**) shows the three dimensional view of the computed trajectory.

Next, we show a second experiment recorded with the IMU DSP-1750 with the set up previously described and shown in Figure 8a. The trajectory has been computed integrating the quasi-error-free turn rate measurements without subtracting the biases or applying updates, and the step detector, step length estimator and vertical displacement estimator proposed in [18,19]. The trajectory might contain errors due to these aforementioned algorithms e.g., errors in the step length or vertical displacement estimation. Nevertheless, the algorithms proposed in this work should work in the presence of these errors and in the presence of drift. The trajectory depicted in Figure 10 includes corners of small and large curvature radii and also stairs out- and inside the Earth Observation Center building of DLR.

**Figure 11:**
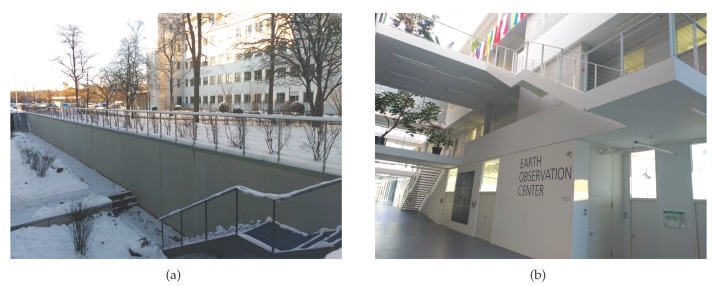
(**a**) shows the three outdoor stairs of the building and (**b**) shows the two indoor stairs.

The start and end point of the walk is located outdoors as the red colored pin of Figure 10a indicates. Table 4 shows the first 9 landmarks automatically detected during the walk described in the following: The pedestrian starts walking leaving the front garden of the building at his left hand. The first described corner is not detected due to its large curvature radius. Therefore, it is not taken into account for the drift computation, as well as the two following curves, since they do not describe a 90${}^{\circ }$ turn. The first detected corner lies on the back garden and is highlighted with a yellow pin in Figure 10a. Then three outdoor stairs to access the basement are detected with a very similar yaw angle (see Table 4), because they are aligned. Their location has been represented with pink placemarks on the back garden. Then, two more corners are detected outdoors until the pedestrian walks into the building. All described indoor corners are detected and also the indoor stairs. In total, 36 landmarks have been detected for the trajectory shown in Figure 10. The first landmark described indoors corresponds to the stairs, which is twice detected due to the landing zone, unlike the case of the outdoors upstairs which also have a landing zone as shown in Figure 11.

The double detection may happen depending on the length of the landing zone of the stairs, being harmless to the drift computation. As Table 4 shows, the yaw angle detected for both sections of the indoor stairs, L7 and L8, is very similar and approximately 180${}^{\circ }$ shifted to the detected direction in which the outdoor stairs, L2, L3 and L4, were described, as expected.

### 3.3. Landmark Associator

Once a landmark has been detected, the association process starts. The first step is to identify whether the current landmark has been previously detected or it is new to the data base. The association is based on the Mahalanobis distance Δ (Equation (4)) between the current landmark and those stored in the data base, and the threshold κ.

**Figure 12:**
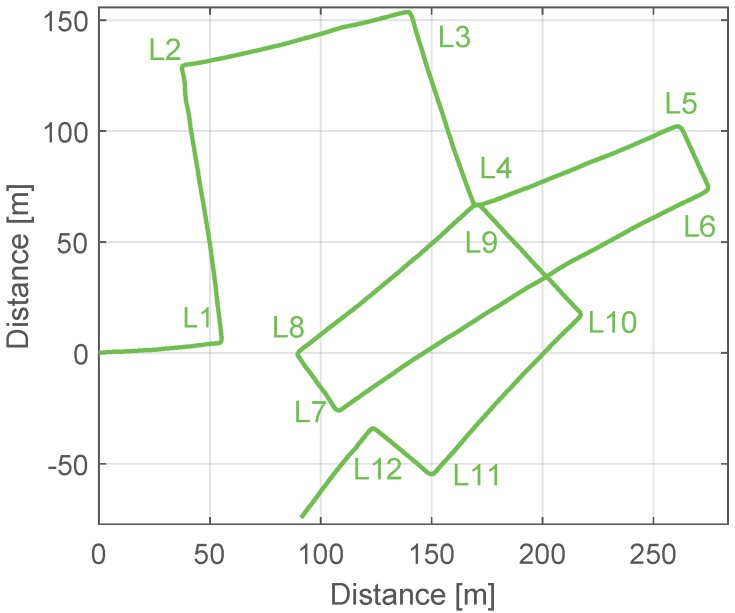
Trajectory computed using the MEMS-quality measurements with a set of biases equal to (0,0,0.05)${}^{\circ }$·s${}^{-1}$. The detected corners are labeled.

First, we analyze the experiment shown in Figure 8b. The red pin represents the start and the end point of the trajectory and the rest of the pins highlight the described corners. Among these corners, the green pins represent the possible associations. We will test the landmark associator with the drifted trajectory, which is represented in Figure 12. As aforementioned, three associations are correct: (L7, L1), (L9, L4) and (L12, L1) or (L12, L7). The information of the detected landmarks was shown in Table 3. The threshold κ is 4.48.

The Mahalanobis distances computed for the landmark L7 are summarized in Table 5. As the table shows, the landmarks (L7, L1) are correctly associated.

The Mahalanobis distances computed for the landmark L9 are summarized in Table 6. As the table shows, the association (L9, L4) is selected as it provides a very small Mahalanobis distance. Indeed, the association is correct.

The Mahalanobis distances computed for the landmark L12 are summarized in Table 7.

The uncertainty of the position and the uncertainty of the yaw angle are ever growing. This leads to multiple association hypotheses. In this case, L12 can also be associated with L8, L9 and L10. The landmark L11 lies under the threshold κ, but the time between detections is not significant for computing the drift, thus it is not taken into account. The correct associations are (L12, L7) or (L12, L1). The drift has been computed using the landmark L7, because it has the smallest Mahalanobis distance. Apart from the correct associations detailed in Table 5, Table 6 and Table 7, the landmarks L8, L10 and L11 generate associations which are not correct. If the position uncertainty of the pedestrian covers a significant part of the trajectory, many hypotheses should be taken into account. The position uncertainty is formed by the yaw uncertainty and the displacement uncertainty, being the first one the main source of positioning error.

Last, the 3D walk recorded in the Earth Observation Center of DLR shown in Figure 10, has also been evaluated. The associations are correctly made taking into account that: first, only the corners corresponding to the same floor are eligible for the potential associations; second, if the stairs are detected in two sections due to the landing zone, both sections are correctly associated when the landmark is re-visited, because the Mahalanobis distance is smaller between both upper parts and both lower parts.

### 3.4. Drift Computation

Next, the drift is computed following Equation (8) taking into account all associations made during the walk. The drift values computed in all associations should be similar, and particularly for the case of the walk shown in Figure 12, it should be constant over time and equal to 0.05${}^{\circ }$·s${}^{-1}$. Table 8 summarizes the automatically computed drift values, measured in ${}^{\circ }$·s${}^{-1}$, for all detected associations.

The main difficulty of computing the drift is to detect a correct yaw angle for the landmarks. Since the sensor is located on the pedestrian’s upper part of the leg, the resulting yaw angle reflects oscillations due to the movement of the hip while walking, which have an amplitude of up to 20${}^{\circ }$. Additionally, the natural walk does not describe exactly the same angle when re-visiting an area, even if the walkable areas are restricted in manmade scenarios. As Table 8 shows, all detected associations yield to similar drift values. The fact that the correct associations, marked in bold, and the wrong associations yield to similar drift values is due to the architecture of the manmade area visited for the walk (see Figure 8), because the allowed walkable directions are restricted to North-South and East-West.

### 3.5. Drift Compensation

**Figure 13:**
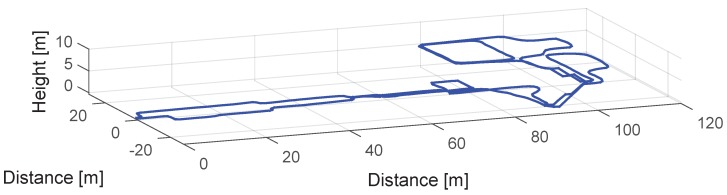
3D view of the trajectory of a 10 min walk at the Deutsches Museum, Munich.

Finally, once the landmarks have been detected, associated and their corresponding drift errors have been computed, $\epsilon_{o}$ is derived following Equation (10). This value is assigned to the z-axis gyroscope’s bias in the post-processing stage. In order to test the proposed drift compensation algorithm with real measurements from medium-cost MEMS IMUs with unknown biases and drift error, we will use the MTw inertial sensors from Xsens. We have recorded a 3D walk at the Deutsches Museum of the city of Munich. The trajectory is shown in Figure 13.

The Deutsches Museum scenario presents a highly perturbed magnetic field, since the visitor walks between metallic pieces of the exhibited ships and airplanes. In such a scenario, the successful estimation of the z-axis gyroscope’s bias based on the magnetic field needs longer than the duration of this 10 min walk (see [2]). For this reason, it is imperative to find alternative methods to compensate the accumulated drift over time, such as the algorithm proposed in this work. It has been decided to dismiss magnetic measurements and only use acceleration and turn rate measurements to compute the 3D trajectory shown in Figure 13 in order to prove the effectiveness of the proposed landmark-based drift compensation algorithm. Therefore, the orientation has been computed with the orientation estimation filter detailed in [2], applying only corrections based on the gravity field. The horizontal and vertical displacement estimation have been computed using the algorithms proposed in [18,19].

The trajectory shown in Figure 13 has been already drift compensated. We have computed $\epsilon_{o}$ following the procedure shown in Section 3.1, resulting in a drift error equal to −0.065${}^{\circ }$·s${}^{-1}$. We do not count with ground truth position within the museum building, thus we start and end the walk at the same point to have a visual indication when the drift has been corrected. The volunteer starts at (0, 0, 0) and walks into the museum until the point (90, −20, 0). Then she takes the stairs to reach the first floor when she describes a trajectory with a "P" shape and then she walks again to the stairs in order to reach the second floor. On the second floor she walks over the rear part of the museum and then she describes twice a rectangular shape corridor. Finally, the volunteer takes the stairs to go back to the starting point in the ground floor.

**Figure 14:**
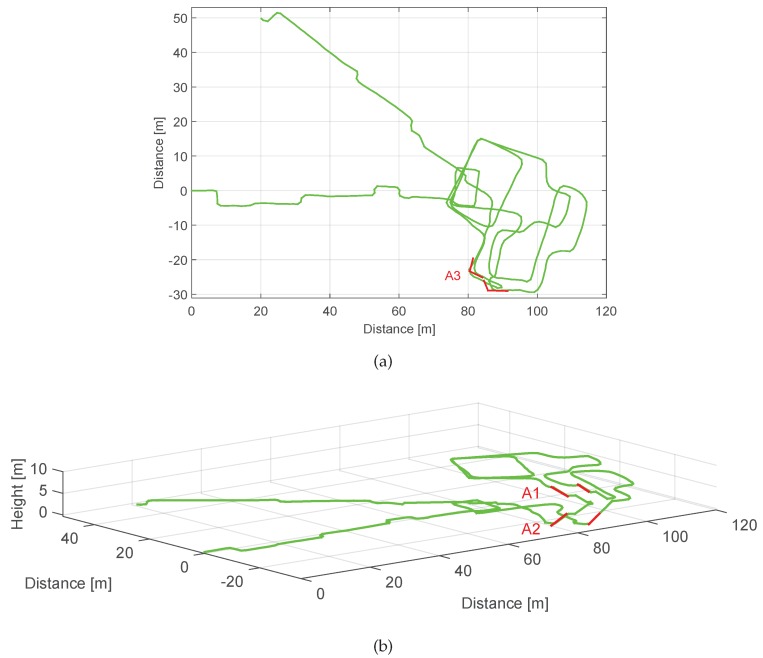
2D and 3D view of the walk recorded at the Deutsches Museum without compensating the drift. The associations are highlighted in the figure: (**a**) includes in red the corner situated on the ground floor and (**b**) includes in red the associations of the staircase.

Figure 14 shows the 2D and 3D view of the walk computed as aforementioned, but without compensating the drift with the algorithm proposed in this work. The effect of the drift is clearly visible, gradually twisting the trajectory in a way that the initial and ending points are almost 54 m away. The associations made by the algorithm we propose in this work have been marked in the figure as A1, A2 and A3.

The stairs connecting the first floor and the second floor, A1, and the stairs connecting the ground floor and the first floor, A2, highlighted in red in Figure 14b have been successfully associated, even though the stairs are in some cases detected in two sections due to the landing zone. Last, one corner on the ground floor at the beginning of the stairs, A3, highlighted in red in Figure 14a has also been associated. The loops described on the third floor lead to two possible corner associations, however, these are automatically disregarded because the time between landmarks is not significant to accumulate drift. Table 9 summarizes the deviation angles δ, the time between associated landmarks Δt and the drift ϵ values computed by the proposed algorithm.

**Figure 15:**
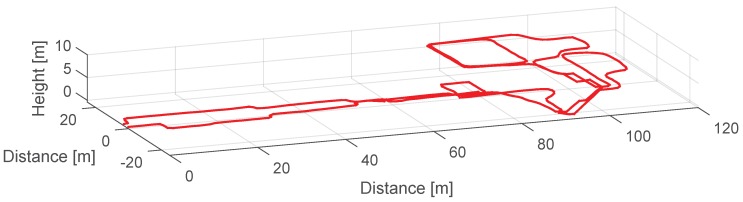
3D trajectory drift compensated with $\epsilon_{o}$ equals to −0.068${}^{\circ }$·s${}^{-1}$.

Using Equation (10) the value $\epsilon_{o}$ is computed being the result equal to −0.068${}^{\circ }$·s${}^{-1}$, which is very similar to the correct drift value −0.065${}^{\circ }$·s${}^{-1}$. Figure 15 shows the resulting trajectory in 3D if the drift error is compensated using the value of −0.068${}^{\circ }$·s${}^{-1}$ computed by the proposed algorithm. We conclude that, thanks to the algorithm proposed in this work, the underlying drift error accumulated over a determined trajectory can be automatically computed and successfully compensated in a post-processing stage. We assume, however, a constant drift error during the whole trajectory. This assumption does not hold for long periods, thus based on the proof of concept demonstrated in this work, the drift should be compensated while the landmarks are detected in order to adapt to drift variations.

## 4. Conclusions

Yaw angle estimations suffer from high accumulated error when using current medium- and low-cost MEMS gyroscopes. This error is called drift and it is mainly composed of biases and other slow changing errors, such as temperature variations. We propose in this work a seamless landmark-based drift compensation algorithm that only uses inertial sensors. This algorithm adds a great value to the state of the art, because the drift error is computed and used by the orientation estimation filter to generate low-drifted yaw angle estimations. The state of the art algorithms are based on applying only position corrections, which do not prevent the yaw angle error growth.

The proposed algorithm requires that landmarks, i.e., stairs and corners, are re-visited allowing trajectories completely or partially overlapped or even with no overlap. We demonstrate that the proposed landmarks can be successfully detected while the pedestrian walks by using only inertial sensors. Errors due to the oscillation of the hip while walking or due to not describing exactly the same landmark trajectory during the re-visit are attenuated by weighting more associations whose elapsed time between landmarks is larger. Associations are decided taking into account the uncertainty of the position, thus if the uncertainty is large due to the accumulated drift error, more than one hypothesis might be possible. The proposed algorithm has been tested with quasi-error-free turn rate measurements with known biases and medium-cost gyroscopes in 3D indoor and outdoor scenarios. We prove that the underlying drift error accumulated over a determined trajectory can be automatically computed and successfully compensated in a post-processing stage. Therefore, with this proof of concept we provide a solid base for an algorithm to compensate the drift online during the walk and so adapt to drift variations.

## Figures and Tables

**Table 1:** Data Base Example.

	Type	Time (s)	Position (m)	Uncertainty (m${}^{2}$)	Yaw (${}^{\circ }$)
L1	0	17.3	(5.12, 2.06, 3.5)	$\left(\begin{array}{ccc} 0.45 & -0.31 & 0\\ -0.31 & 0.41 & 0\\ 0 & 0 & 0.02 \end{array}\right)$	(24.5, −80.4)
L2	1	59.6	(7.11, −1.03, 2)	$\left(\begin{array}{ccc} 0.62 & -0.42 & 0\\ -0.42 & 0.89 & 0\\ 0 & 0 & 0.28 \end{array}\right)$	90.3

**Table 2:** Landmark Information.

	Type	Time (s)	Position (m)	Uncertainty (m${}^{2}$)	Yaw (${}^{\circ }$)
L4	0	432.3	-	-	(−66.7, 23.1)
L9	0	870.1	-	-	(−314.9, −403.7)

**Table 3:** Data Base Generated for the Drifted Trajectory Depicted in Figure 9b.

	Type	Time (s)	Position (m)	Uncertainty (m${}^{2}$)	Yaw (${}^{\circ }$)
L1	0	131.6	(55.63, 5.84, 0)	-	(7.62, 86.76)
L2	0	248.0	(38.69, 127.17, 0)	-	(102.38, 22.30)
L3	0	348.2	(137.02, 148.71, 0)	-	(14.94, −63.14)
L4	0	436.9	(167.98, 67.35, 0)	-	(−69.27, 20.72)
L5	0	525.1	(253.98, 96.92, 0)	-	(23.03, −64.40)
L6	0	552.6	(260.91, 71.81, 0)	-	(−63.93, −149.98)
L7	0	744.2	(106.66, −15.65, 0)	-	(−140.73, −237.35)
L8	0	774.2	(95.31, 7.58, 0)	-	(−233.36, −322.20)
L9	0	873.7	(168.52, 63.90, 0)	-	(−318.15, −403.04)
L10	0	936.0	(205.98, 18.92, 0)	-	(−401.74, −495.63)
L11	0	1035.4	(144.98, −40.57, 0)	-	(−490.09, −579.07)
L12	0	1068.9	(122.27, −26.70, 0)	-	(−574.61, −487.75)

**Table 4:** Part of the Data Base Generated for the Trajectory Depicted in Figure 10.

	Type	Time (s)	Position (m)	Uncertainty (m${}^{2}$)	Yaw (${}^{\circ }$)
L1	0	169.3	(−46.07, 73.55, 0)	-	(178.74, 268.70)
L2	1	264.4	(−34.35, 24.10, −0.99)	-	453.74
L3	1	306.0	(−33.78, 59.52, −2.99)	-	450.62
L4	1	314.8	(−33.41, 63.55, −0.99)	-	449.61
L5	0	327.4	(−30.41, 72.50, −0.33)	-	(454.41, 364.42)
L6	0	347.99	(−10.27, 70.31, −0.33)	-	(368.37, 272.85)
L7	1	372.1	(−8.55, 47.43, 0.33)	-	272.19
L8	1	380.2	(−8.95, 43.66, 1.99)	-	269.47
L9	0	388.0	(−11.56, 40.65, 2.99)	-	(268.49, 185.40)

**Table 5:** Mahalanobis Distances Computed for the Association of Corner L7 of Figure 12.

	L1	L2	L3	L4	L5	L6
Δ	**4.33**	10.26	11.05	6.70	12.21	11.28

**Table 6:** Mahalanobis Distances Computed for the Association of Corner L9 of Figure 12.

	L1	L2	L3	L4	L5	L6	L7	L8
Δ	9.75	8.66	5.03	**0.38**	5.70	5.37	5.02	4.55

**Table 7:** Mahalanobis Distances Computed for the Association of Corner L12 of Figure 12.

	L1	L2	L3	L4	L5	L6	L7	L8	L9	L10	L11
Δ	**4.16**	8.15	8.18	4.74	8.55	7.92	**0.79**	1.55	3.82	3.50	0.76

**Table 8:** Drift Values Measured in ${}^{\circ }$·s${}^{-1}$, of the Walk of Figure 12.

(L7, L1)	(L8, L1)	(L9, L4)	(L10, L4)	(L11, L7)	(L12, L7)
0.055	0.054	0.054	0.051	0.049	0.055

**Table 9:** Associations of the Walk Shown in Figure 14.

	δ (${}^{\circ }$)	Δt (s)	ϵ (${}^{\circ }$·s${}^{-1}$)
A1	−17	186.1	**−0.091**
A2	−18.3	295.1	**−0.062**
A3	−19.1	307.6	**−0.062**
